# Endothelial Dysfunction: From a Pathophysiological Mechanism to a Potential Therapeutic Target

**DOI:** 10.3390/biomedicines10010078

**Published:** 2021-12-31

**Authors:** Pasquale Ambrosino, Guido Grassi, Mauro Maniscalco

**Affiliations:** 1 Istituti Clinici Scientifici Maugeri IRCCS, Cardiac Rehabilitation Unit of Telese Terme Institute, 82037 Telese Terme, Benevento, Italy; 2 Clinica Medica, Department of Medical Sciences, University of Milano-Bicocca, 20126 Milan, Italy; guido.grassi@unimib.it; 3 Istituti Clinici Scientifici Maugeri IRCCS, Pulmonary Rehabilitation Unit of Telese Terme Institute, 82037 Telese Terme, Benevento, Italy

The endothelium is considered the largest organ of the body, composed of a monolayer of endothelial cells (ECs) lining the interior surface of blood and lymphatic vessels [1]. ECs are able to respond to a number of humoral and hemodynamic stimuli by producing a wide range of mediators regulating vascular tone, cellular adhesion, coagulation, smooth muscle cell proliferation and vessel wall inflammation [1]. Given its biological properties, nitric oxide (NO) is one of the most important endothelium-derived mediators [2]. NO is a soluble gas synthetized from the amino acid L-arginine by the calcium/calmodulin-dependent NO synthase (NOS) [2]. However, a number of both NO-dependent and NO-independent pathways have been called into question to explain the homeostatic functions of the endothelium.

Despite the different phenotypic characteristics displayed by ECs in various organs and tissues, endothelial dysfunction shares some common features, such as reduced vasodilation, inflammation, oxidative stress and a prothrombotic state [3]. Thus, the presence of a dysfunctional endothelium has been identified as a key and early pathogenic mechanism in many acute and chronic diseases, including infections, cancer, chronic obstructive pulmonary disease, heart failure, pulmonary hypertension, and metabolic and autoimmune disorders [4,5]. Most importantly, endothelial dysfunction contributes to atherosclerotic plaque initiation and progression, thus being considered the earliest stage of most cardiovascular (CV) diseases [6]. More recently, growing evidence suggested that endothelial dysfunction may be the common pathogenic background of most manifestations of coronavirus disease 2019 (COVID-19) in the acute phase and in the rehabilitation setting, since ECs are a preferential target of the novel severe acute respiratory syndrome coronavirus 2 (SARS-CoV-2) [7].

Given the different clinical and laboratory methods for monitoring endothelial dysfunction and considering its systemic nature and reversibility in early stages, this condition has been proposed as an attractive therapeutic target in many clinical conditions, with a potential emerging role for specific pharmacological interventions and tailored exercise-based rehabilitation strategies [8,9].

In this Special Issue, we aimed to collect some original research articles and reviews on the mechanisms and diagnosis as well as the prognostic and therapeutic implications of endothelial dysfunction as a biomarker of inflammation, oxidative stress and vascular disease.

Thus, a number of high-quality reviews focusing on different aspects of the complex interplay between endothelial dysfunction, inflammation and CV risk were published, pointing out the importance of some molecular mechanisms. The Notch pathway emerged as a master regulator of angiogenesis, with an important role in transducing the signals provided by the blood shear stress to the endothelium [10]. The cardioprotective effect of adropin, a hepatic peptide involved in glucose metabolism acting via the Notch signaling pathway, was discussed in another review article [11]. Instead, Theofilis et al. provided an exhaustive description of the inflammatory mechanisms contributing to endothelial dysfunction, pointing out the emerging role of neutrophil extracellular traps and NLR family pyrin domain containing 3 (NLRP3) inflammasome [12]. The crosstalk between ECs and the sympathetic nervous system was discussed in another article, with particular emphasis on its role in the pathogenesis of essential hypertension and congestive heart failure [13]. Finally, Salvatore et al. analyzed the CV protection mechanisms of the sodium glucose transporter 2 (SGLT2) inhibition in type 2 diabetes, discussing the role of gliflozins in modulating endothelial function through the attenuation of oxidative stress and inflammation [14].

Some preclinical and functional studies also fell within the scope of this Special Issue. In an intruding article by Kan et al. [15], the effect of blue light irradiation at a 453-nanometer wavelength on human umbilical vein ECs was explored. The authors showed that low-fluence blue light irradiation activated the angiogenic pathways, such as the vascular endothelial growth factor (VEGF) signaling pathway, thus promoting cell viability, migration and angiogenesis. In contrast, high-fluence illumination caused the opposite effect on those activities by up-regulating pro-apoptotic signaling cascades, including ferroptosis, necroptosis and the p53 signaling pathways. Two articles [16,17] focused on the prognostic and pathogenetic role of endothelial dysfunction in systemic sclerosis (SSc). Lo Gullo et al. [16] documented that SSc patients exhibit significantly higher endocan levels as compared with healthy controls, with a direct correlation between this laboratory marker of endothelial dysfunction and the severity of pulmonary impairment. Of interest, they did not document any significant difference in endothelial progenitor cells (EPCs) levels between cases and controls, while Pulito-Cueto et al. [17] reported an opposite finding. These apparently contrasting results, potentially due to the different methods for EPCs’ characterization and patient inclusion criteria, further support the need for large preclinical and laboratory studies evaluating the prognostic role of EPCs levels and function in different clinical settings. Two further studies [18,19] in the Special Issue focused on endothelial dysfunction in COVID-19. In particular, Macor et al. [18] performed immunofluorescence analyses of autopsy specimens of lungs, kidney and liver from 12 COVID-19 patients who died of acute respiratory failure. Interestingly, they found complement deposition on vascular endothelium of all the analyzed specimens. In another study [19], a significant improvement in the endothelium-dependent flow-mediated dilation (FMD) of convalescent COVID-19 patients was documented after multidisciplinary rehabilitation, with a potential reduction in the CV risk. Moreover, a direct and persistent correlation between the severity of pulmonary and vascular disease was reported, providing preliminary information on the potential usefulness of exercise-based rehabilitation and strategies targeting endothelial function. Finally, in a large clinical study [20] on 653 Caucasian never-treated hypertensives, endothelial dysfunction showed a prognostic role in predicting the onset of diabetes and future CV events.

Other pertinent studies [21,22,23] have been published in this Special Issue, all of which contribute to providing an interesting insight into the molecular mechanisms of endothelial dysfunction and its role as a biomarker of inflammation, oxidative stress and vascular disease. The prognostic and therapeutic implications of endothelial dysfunction have been analyzed in both review and original articles, with intriguing new findings and potentially relevant repercussions in clinical practice and future research. The studies published in this Special Issue support the analysis of different measures of endothelial function as a useful tool in the follow-up of several clinical conditions. This could help establish comprehensive and personalized prevention, interventional and rehabilitation strategies aimed at reducing disease progression, CV risk and subsequent disability. More translational and laboratory research is needed to fully elucidate the homeostatic functions of the endothelium, thus clarifying the role of endothelial dysfunction in various clinical conditions.

## Data Availability

No datasets were generated or analysed during the current study.

## References

[1] Vane J.R., Anggard E.E., Botting R.M. (1990). Regulatory functions of the vascular endothelium. N. Engl. J. Med..

[2] Tare M., Parkington H.C., Coleman H.A., Neild T.O., Dusting G.J. (1990). Hyperpolarization and relaxation of arterial smooth muscle caused by nitric oxide derived from the endothelium. Nature.

[3] Feletou M., Vanhoutte P.M. (2006). Endothelial dysfunction: A multifaceted disorder (The Wiggers Award Lecture). Am. J. Physiol. Heart Circ. Physiol..

[4] Ambrosino P., Lupoli R., Tortora A., Cacciapuoti M., Lupoli G.A., Tarantino P., Nasto A., Di Minno M.N. (2016). Cardiovascular risk markers in patients with primary aldosteronism: A systematic review and meta-analysis of literature studies. Int. J. Cardiol..

[5] Ambrosino P., Lupoli R., Iervolino S., De Felice A., Pappone N., Storino A., Di Minno M.N.D. (2017). Clinical assessment of endothelial function in patients with chronic obstructive pulmonary disease: A systematic review with meta-analysis. Intern. Emerg. Med..

[6] Zeiher A.M., Drexler H., Wollschlager H., Just H. (1991). Modulation of coronary vasomotor tone in humans. Progressive endothelial dysfunction with different early stages of coronary atherosclerosis. Circulation.

[7] Ambrosino P., Papa A., Maniscalco M., Di Minno M.N.D. (2021). COVID-19 and functional disability: Current insights and rehabilitation strategies. Postgrad. Med. J..

[8] Cornelissen V.A., Onkelinx S., Goetschalckx K., Thomaes T., Janssens S., Fagard R., Verhamme P., Vanhees L. (2014). Exercise-based cardiac rehabilitation improves endothelial function assessed by flow-mediated dilation but not by pulse amplitude tonometry. Eur. J. Prev. Cardiol..

[9] Kourek C., Alshamari M., Mitsiou G., Psarra K., Delis D., Linardatou V., Pittaras T., Ntalianis A., Papadopoulos C., Panagopoulou N. (2021). The acute and long-term effects of a cardiac rehabilitation program on endothelial progenitor cells in chronic heart failure patients: Comparing two different exercise training protocols. Int. J. Cardiol. Heart Vasc..

[10] Fortini F., Vieceli Dalla Sega F., Marracino L., Severi P., Rapezzi C., Rizzo P., Ferrari R. (2021). Well-Known and Novel Players in Endothelial Dysfunction: Updates on a Notch(ed) Landscape. Biomedicines.

[11] Bozic J., Kumric M., Ticinovic Kurir T., Males I., Borovac J.A., Martinovic D., Vilovic M. (2021). Role of Adropin in Cardiometabolic Disorders: From Pathophysiological Mechanisms to Therapeutic Target. Biomedicines.

[12] Theofilis P., Sagris M., Oikonomou E., Antonopoulos A.S., Siasos G., Tsioufis C., Tousoulis D. (2021). Inflammatory Mechanisms Contributing to Endothelial Dysfunction. Biomedicines.

[13] Quarti-Trevano F., Seravalle G., Grassi G. (2021). Clinical Relevance of the Sympathetic-Vascular Interactions in Health and Disease. Biomedicines.

[14] Salvatore T., Caturano A., Galiero R., Di Martino A., Albanese G., Vetrano E., Sardu C., Marfella R., Rinaldi L., Sasso F.C. (2021). Cardiovascular Benefits from Gliflozins: Effects on Endothelial Function. Biomedicines.

[15] Kan K., Mu Y., Bouschbacher M., Sticht C., Kuch N., Sigl M., Rahbari N., Gretz N., Pallavi P., Keese M. (2021). Biphasic Effects of Blue Light Irradiation on Human Umbilical Vein Endothelial Cells. Biomedicines.

[16] Lo Gullo A., Mandraffino G., Rodriguez-Carrio J., Scuruchi M., Sinicropi D., Postorino M., Morace C., Giuffrida C., Sciortino D., Gallizzi R. (2021). Endocan and Circulating Progenitor Cells in Women with Systemic Sclerosis: Association with Inflammation and Pulmonary Hypertension. Biomedicines.

[17] Pulito-Cueto V., Remuzgo-Martinez S., Genre F., Atienza-Mateo B., Mora-Cuesta V.M., Iturbe-Fernandez D., Lera-Gomez L., Perez-Fernandez R., Prieto-Pena D., Portilla V. (2021). Endothelial Progenitor Cells: Relevant Players in the Vasculopathy and Lung Fibrosis Associated with the Presence of Interstitial Lung Disease in Systemic Sclerosis Patients. Biomedicines.

[18] Macor P., Durigutto P., Mangogna A., Bussani R., De Maso L., D’Errico S., Zanon M., Pozzi N., Meroni P.L., Tedesco F. (2021). Multiple-Organ Complement Deposition on Vascular Endothelium in COVID-19 Patients. Biomedicines.

[19] Ambrosino P., Molino A., Calcaterra I., Formisano R., Stufano S., Spedicato G.A., Motta A., Papa A., Di Minno M.N.D., Maniscalco M. (2021). Clinical Assessment of Endothelial Function in Convalescent COVID-19 Patients Undergoing Multidisciplinary Pulmonary Rehabilitation. Biomedicines.

[20] Maio R., Suraci E., Caroleo B., Politi C., Gigliotti S., Sciacqua A., Andreozzi F., Perticone F., Perticone M. (2021). New-Onset Diabetes, Endothelial Dysfunction, and Cardiovascular Outcomes in Hypertensive Patients: An Illness-Event Model Analysis. Biomedicines.

[21] Meiners J., Jansen K., Gorbokon N., Büscheck F., Luebke A.M., Kluth M., Hube-Magg C., Höflmayer D., Weidemann S., Fraune C. (2021). Angiotensin-Converting Enzyme 2 Protein Is Overexpressed in a Wide Range of Human Tumour Types: A Systematic Tissue Microarray Study on >15,000 Tumours. Biomedicines.

[22] Little P.J., Askew C.D., Xu S., Kamato D. (2021). Endothelial Dysfunction and Cardiovascular Disease: History and Analysis of the Clinical Utility of the Relationship. Biomedicines.

[23] Berenyiova A., Bernatova I., Zemancikova A., Drobna M., Cebova M., Golas S., Balis P., Liskova S., Valaskova Z., Krskova K. (2022). Vascular Effects of Low-Dose ACE2 Inhibitor MLN-4760—Benefit or Detriment in Essential Hypertension?. Biomedicines.

